# Prospective randomised controlled trial of Algisite™ M, Cuticerin™, and Sorbact® as donor site dressings in paediatric split-thickness skin grafts

**DOI:** 10.1186/s41038-018-0135-y

**Published:** 2018-11-27

**Authors:** Craig A. McBride, Roy M. Kimble, Kellie A. Stockton

**Affiliations:** 1 Pegg Leditschke Children’s Burns Centre, Queensland Children’s Hospital, Children’s Health Queensland Hospital and Health Service, South Brisbane, Queensland Australia; 2 Centre for Children’s Burns and Trauma Research, Centre for Children’s Health Research, University of Queensland, South Brisbane, Australia; 3 Discipline of Paediatrics and Child Health, School of Medicine, University of Queensland, St Lucia, Queensland Australia

**Keywords:** Split-thickness skin graft, Donor site wound, Paediatric, Burns, Alginate, Algisite™ M, Cuticerin™, Sorbact®

## Abstract

**Background:**

This is a parallel three-arm prospective randomised controlled trial (RCT) comparing Algisite™ M, Cuticerin™, and Sorbact® as donor site dressings in paediatric split-thickness skin grafts (STSG). All three were in current use within the Pegg Leditschke Children’s Burn centre (PLCBC), the largest paediatric burns centre in Queensland, Australia. Our objective was to find the best performing dressing, following on from previous trials designed to rationalise dressings for the burn wound itself.

**Methods:**

All children for STSG, with thigh donor sites, were considered for enrolment in the trial. Primary outcome measures were days to re-epithelialisation, and pain. Secondary measures were cost, itch, and scarring at 3 and 6 months. Patients and parents were blinded to group assignment. Blinding of assessors was possible with the dressing *in situ*, with partial blinding following first dressing change. Blinded photographic assessments of re-epithelialisation were used. Scar assessment was blinded. Covariates for analysis were sex, age, and graft thickness (as measured from a central biopsy).

**Results:**

There were 101 patients randomised to the Algisite™ M (33), Cuticerin™ (32), and Sorbact® (36) arms between April 2015 and July 2016. All were analysed for time to re-epithelialisation. Pain scores were not available for all time points in all patients. There were no significant differences between the three arms regarding pain, or time to re-epithelialisation. There were no significant differences for the secondary outcomes of itch, scarring, or cost. Regression analyses demonstrated faster re-epithelialisation in younger patients and decreased donor site scarring at 3 and 6 months with thinner STSG. There were no adverse effects noted.

**Conclusions:**

There are no data supporting a preference for one trial dressing over the others, in donor site wounds (DSW) in children. Thinner skin grafts lead to less donor site scarring in children. Younger patients have faster donor site wound healing.

**Trial registration:**

Australia and New Zealand Clinical Trials Register (ACTRN12614000380695).

Royal Children’s Hospital Human Research Ethics Committee (HREC/14/QRCH/36).

University of Queensland Medical Research Ethics Committee (#2014000447).

## Background

Surgeons deliberately create wounds in order to treat patients. The donor site wound (DSW) of a split-thickness skin graft (STSG) is one such example. Given the size of these injuries, it is perhaps surprising that more effort has not been devoted to minimising their morbidity [1]. There is more literature on the treatment of the burn wound itself than there is on treatment of the iatrogenic DSW. Opinion on the best dressing for a DSW often stems from eminence, rather than evidence.

The definition of the ‘best’ DSW dressing has been the subject of some debate, and there are a number of studies investigating clinician preferences. An ideal DSW dressing does not adhere to the wound, thus facilitating removal. DSW dressings should be pain-free, reduce blood loss, and be changed infrequently, ideally, not until the wound has re-epithelialised [2–5]. DSWs are recognised as often being more painful than the STSG recipient site [6]. Most studies focus on short-term effects, such as pain and time to re-epithelialisation. Some of these effects may be synergistic. For example, there are data in children suggesting decreased pain leads to more rapid re-epithelialisation [7–9]. Few studies have investigated the longer-term effects of DSW dressings, such as itch or scarring.

The Pegg Leditschke Children’s Burns Centre (PLCBC) treats over 1000 children with new burns annually. Approximately 10% of these children will require a STSG [10]. The PLCBC was already using the three different DSW dressings in this randomised controlled trial (RCT), according to surgeon preference and with no individual dressing predominant. Our objective was to determine the best performing dressing. Our hypothesis was that one DSW dressing would be superior to the others, in aggregate, with respect to the short- and longer-term outcomes defined below. Anecdotally, the dressings perform similarly in the early phases of healing (i.e., during skin re-epithelialisation), so measures of scarring were included, to unmask any potential long-term DSW dressing superiority.

## Methods

### Trial design

This study was a single-centre three-arm parallel group prospective, RCT. The trial was registered with the Australia and New Zealand Clinical Trials Register (ACTRN12614000380695) [11]. It was approved by the Royal Children’s Hospital Human Research Ethics Committee (HREC/14/QRCH/36), and the University of Queensland Medical Research Ethics Committee (#2014000447). The methodology was specified in advance and documented in a published protocol [12]. The methods are summarised according to the revised Consolidated Standards for Reporting Trials (CONSORT) statement [13]. The trial was largely completed as per published protocol [12]. Deviations from the published protocol are highlighted, and their reasons outlined, below.

### Participants

The PLCBC treats patients under 16 years of age. From trial commencement, all consecutive children undergoing STSG were considered for inclusion. Exclusion criteria included non-English-speaking parents/caregivers; children with a known cognitive impairment; children under the care of, or investigation by, the Department of Communities, Child Safety and Disability Services. These exclusions were chosen because of difficulties with subsequent recording of outcomes measures, since many of these were parent/carer reports rather than staff or clinician outcome reports only. Only thigh DSWs were included.

Demographic data and outcome measures were collected in the burns clinic both pre- and post-grafting. Intervention data were collected intraoperatively.

### Intervention

Prior to the trial there were three DSW dressings in use within the PLCBC; hence, the three arms chosen:

1. Algisite™ M, an alginate dressing (Smith & Nephew, Hull, UK),
2. Cuticerin™, a smooth acetate gauze impregnated with water repellent ointment (petrolatum, paraffin and Eucerite®) (Smith & Nephew, Hull, UK), and
3. Sorbact®, a gauze mesh coated with a dialkylcarbamoyl chloride (DACC; a synthetic fatty acid derivative) and amorphous hydrogel (Abigo Medical AB, Gothenburg, Sweden) [14].

Previously recruited and enrolled patients were randomised intraoperatively immediately prior to definitive DSW dressing application. Where STSGs were harvested from bilateral thighs, a second randomisation was permitted. Each wound constituted its own study entity and was analysed separately. Trial dressings were applied directly to the wound and then covered with Allevyn™ and a Hypafix™ securement dressing (Smith & Nephew, Hull, UK).

The STSG was harvested in all cases using a pneumatic dermatome (Zimmer Inc., Warsaw, IN, USA) at a depth of 0.007 in., our local default setting. The ‘swipe number’ of the dermatome blade in use at the time, the donor site, and the surgeon harvesting the graft were all recorded. A small lentiform biopsy was taken from the centre of each graft. This was subsequently blocked, cut, and measured for graft thickness in order to provide an objective measurement of the thickness of the graft [15].

Local anaesthetic (bupivacaine 0.25% with epinephrine 1:200000 AstraZeneca Pty Ltd., North Ryde, New South Wales 2113, Australia) was applied directly to the wound at a dose of 2–2.5 mg/kg. Post-harvest topical application, rather than infiltration, was chosen to further standardise the DSW. In longer cases, with larger and/or multiple DSWs, some sites were temporarily dressed with epinephrine/saline-soaked gauze (1 mg/1000 mL NaCl), with or without local pressure, to facilitate haemostasis. The DSW was formally dressed following dressing of the STSG site.

The majority of patients were sent home that day, following a 4-h review and initial scoring for pain and itch. A follow-up phone call at 24 h replicated these questions. Further scores were taken just prior to, and 2 min following, dressing changes. The first dressing change was approximately 1-week post-STSG (nearest weekday), which is our usual pre-trial practice. The DSW dressing was changed first to avoid confounding with pain from the STSG dressing change. The remainder of the protocol has previously been published [12].

### Primary outcome measures

#### Pain

Assessments for pain and distress occurred: (1) 4 h following completion of the STSG operation; (2) 24 h following STSG; (3) prior to dressing removal, and 2 min after dressing removal, for each dressing change until re-epithelialised. Parents/caregivers, nursing staff, and older children (above 3 years) recorded assessments at each of the above time points. Analgesic, sedative, and/or anti-itch medication use was also recorded.

Nursing staff assessing pain and distress used the face, legs, activity, cry, consolability (FLACC) scale. Parents used an 11-point (0–10) numeric rating scale (NRS). Children over 3 years used the Revised Faces Pain Scale (FPS-R).

#### Days to re-epithelialisation

The endpoint was at least 95% re-epithelialisation of the DSW. This was calculated in a number of ways: (1) clinical judgement by the treating consultant, (2) photography using a three-dimensional (3D) camera (3D LifeViz system) and analysis on the Dermapix™ software (Quantificare, Cedex, France), and (3) blinded review of photographs. DSW assessment took place only during routine planned dressing changes. Three experienced burns surgeons assessed photographs of each DSW to determine at which dressing change the wound was re-epithelialised. Surgeons were blinded to the dressing used, and a consensus opinion determined definitive days to re-epithelialisation for each DSW. This was used for subsequent statistical analyses. Previous studies have demonstrated good inter- and intra-rater reliability when assessing wound re-epithelialisation using photographs when compared to clinical assessments [16].

### Secondary outcome measures

#### Itch

Itch was scored at the same time points as pain, using the Itch Man Scale. This has been validated in children [17]. Itch was further evaluated as a component of the Patient and Observer Scar Assessment Scale (POSAS) when DSW scars were assessed at 3 and 6 months.

#### Donor site wound scarring and appearance

This was assessed at 3- and 6-month reviews. Multiple methods were used to assess scars: (1) ultrasound measurement of scar thickness, (2) digital 3D photography with edge marking to determine computer mapped areas of scarring, and (3) the POSAS, this latter used as a global rating for DSW scarring [18, 19]. The POSAS has shown superior performance in a systematic review over other scar assessment scales [20]. Both parent and patient were asked, where possible. There are reliability data supporting the use of this scale in children above the age of 6 years [21]. Below this age, the parent’s opinion was sought as a proxy for the child.

#### Ease of dressing

Ease of dressing was originally included in the protocol, using a 5-point (0–4) NRS. This was subsequently abandoned early in the trial as it was confounded by size of the DSW area and by the levels of anxiety being exhibited by patients and/or carers at the time of removal.

#### Cost

Cost was assessed using hospital pricing for the dressings used. Additional visits, where required, were also included in the cost analysis. Routine visits were not costed since these are a standard part of care for all arms. A cost-utility analysis was not possible in this trial. The site and nature of the burn wound itself could not be isolated from the DSW in order to perform a cost-utility analysis on the DSW alone.

### Sample size

Sample size was calculated from primary outcomes. A minimum clinically important difference (MCID) of 3 days was chosen. This would represent one or more fewer dressing changes for the patient, given dressings were changed every 3 days after the first week. The standard deviation (SD) on DSW re-epithelialisation, from our clinical database, is 4 days. With 80% power, and an alpha of 0.05, 28 participants were required in each arm. This sample size was also adequate to determine an MCID of 2 (SD 2.5) in pain scores using FLACC and the NRS. There were no interim analyses performed.

### Randomisation

The randomisation sequence was generated using an online program (http://stattrek.com/), with unrestricted simple randomisation in a 1:1:1 ratio. Allocation concealment was via the use of sequentially numbered, sealed opaque envelopes pre-prepared by a third party. Envelopes were only opened in the operating theatre after the STSG had been harvested and immediately before application of the DSW dressing. The sequence was generated by one of the researchers (KS). Participants were enrolled by the study authors and by research assistants. In cases where one of the surgeons (CAM or RMK) was the responsible clinician, recruitment and enrolment were performed by another researcher. Participants had been previously enrolled in the order in which a decision for grafting was made. The nature of operating list bookings in our institution meant the order of recruitment was not the same as the order of randomisation, separating recruitment from randomisation. Randomisation continued until each of the trial arms had a minimum of 28 participants reaching primary endpoints.

### Blinding

Full blinding was not possible for all aspects of this study. Each DSW dressing had the same secondary dressing applied, concealing the trial dressing from assessors until the first dressing change. Researchers not present in the theatre assessed outcome measures until the first dressing change. Participants and care providers, as well as assessors, were therefore blinded to dressing.

At the first dressing change, the trial dressing was revealed, since all have a distinctive appearance on removal of the secondary dressing. From that point blinding to pain scores was not possible in the clinic setting, as we were not able to use different staff for each dressing change.

Partial blinding was possible regarding the other primary outcome of re-epithelialisation. In clinic, the surgeon assessing the wound was not blinded to the dressing used. Cuticerin™ and Sorbact® both leave a partial imprint of the weave in the early phases of re-epithelialisation, making it difficult to distinguish between the two dressings. Algisite M™ is often difficult to completely remove from the wound. Three surgeons (CAM, RMK, one other) subsequently viewed and scored digital photographs of the wound at each dressing change. These assessors were partially blinded to which arm of the trial the patient was in. Full blinding was not possible here, as surgeons occasionally recognised their own donor sites, or recognised imprints or dressing remnants remaining on the DSW. The photographic assessment was taken as the definitive assessment in an effort to overcome bias.

For longer-term outcome measures, such as scarring, full blinding was possible since assessors were blinded to the nature of the original DSW dressing.

A number of secondary outcomes, such as itch, were partially blinded as above. Scar appearance assessors, other than patient and parent, were also blinded to the DSW dressing used. Scar assessment was performed by trained research staff, and not by surgeons or trial investigators.

### Statistical analysis

Statistical analyses were conducted using SPSS for Mac version 23 (IBM Corporation, Armonk, New York, USA) and Stata version 15 (Statcorp LLC, College Station, TX, USA). Demographic data were analysed through descriptive statistics and between-groups analyses using analysis of variance (ANOVA) and/or non-parametric equivalents where appropriate. Generalised linear models, estimating variance appropriately for repeated measures where required, were used to determine whether there were differences between groups in primary and secondary outcomes. Changes in the intervention effects were examined with thickness of STSG, sex, and age of potential interest. Data were analysed on an intention-to-treat and on a per-protocol basis. Intention-to-treat analysis was the primary approach for this trial. Two-tailed tests were used, with a *p* value of < 0.05 considered significant.

#### Primary outcome measures

A negative binomial regression model was used for days to DSW re-epithelialisation. Elements considered a priori to be of potential interest, in addition to the trial dressings, were as follows: depth of skin graft harvested, sex, and age of the patient [15].

Multilevel generalised linear mixed-effects modelling with a log link function and gamma distribution was used to determine differences in pain between treatment groups at time points stated above.

#### Secondary outcome measures

Secondary outcome measures were analysed using the same methods as above.

#### Subgroup analyses

Thickness of STSG and ease of removal were considered a priori to be of potential interest in examining changes in intervention effects, along with the sex and age of the patient.

### Discontinuation/adverse events

Data were collected and included up to the point where patients discontinued or had an adverse event. If an adverse event occurred, the leading consultant was permitted to break protocol and treat the patient as he or she saw appropriate, including the use of antimicrobial and/or nanocrystalline silver dressings applied to the DSW.

## Results

### Sample and demographic characteristics

**Fig. 1:**
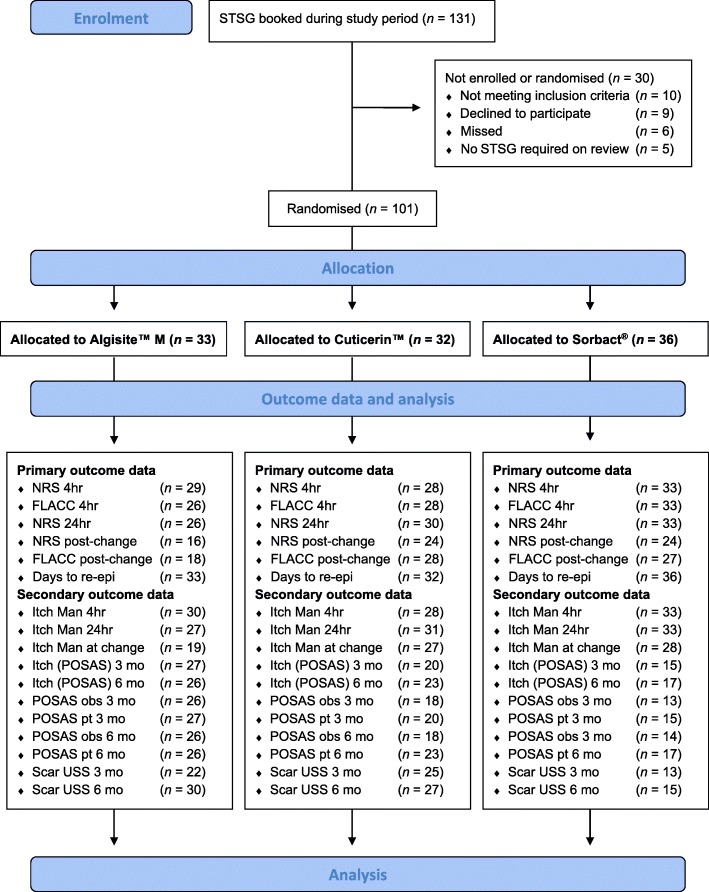
Consolidated Standards for Reporting Trials (CONSORT) diagram for Donor Site Wounds Trial. CONSORT diagram of patients enrolled, and numbers available for outcomes measured. *STSG* split-thickness skin graft, *NRS* numeric rating scale, *FLACC* face, legs, activity, cry, consolability scale, *re-epi* re-epithelialisation, *mo* months, *POSAS* patient observer scar assessment scale, *USS* ultrasound scan, *obs* observer, *pt* patient

Figure 1 demonstrates the Consolidated Standards for Reporting Trials (CONSORT) diagram for this trial [13]. A total of 131 children were booked for STSGs over the course of the trial, 101 of whom (77.1%) were randomised into the trial. Primary outcome data were available for at least 28 patients in each arm on 14 July 2016, at which time recruitment ceased as the number necessary to power the trial had been reached.

The trial itself closed 1 year later, following the final scar assessments. The median size of the DSW was 32.6 cm^2^ (interquartile range (IQR) 14.2–56.7 cm^2^). There were differences between the groups at baseline. These inter-arm differences were accounted for by including age at surgery, sex, and DSW thickness and area as covariates in subsequent analyses—as per the pre-published protocol (Table 1) [12].

**Table 1:** Demographic characteristics of Algisite™ M, Cuticerin™, Sorbact®, including time to ≥ 95% re-epithelialisation. Inter-arm differences included as co-variates in subsequent analyses (as per protocol)

Demographic characteristics of study arms				
	Algisite™ M (*n* = 33) median (IQR)	Cuticerin™ (*n* = 32) median (IQR)	Sorbact® (*n* = 36) median (IQR)	*P* value
Age (years)	5.12 (1.62–8.11)	5.14 (2.18–9.17)	3.70 (1.60–9.49)	0.97
% Males	42.6	60.9	47.9	0.18
Donor site area (cm^2^)	37.5 (16.0–57.8)	30.6 (11.9–71.0)	23.3 (9.6–43.4)	0.034
Median STSG thickness in micrometre (central biopsy)	212.8 (155.5–303.5)	146.9 (115.6–208.0)	171.2 (148.6–234.4)	0.015
Days to re-epithelialisation	7 (7–9)	7 (7–9)	10 (7–11)	0.025

*IQR* interquartile range, *STSG* split-thickness skin graft

### Primary outcome measures

#### Re-epithelialisation

Three surgeons independently scored photographs for re-epithelialisation (95% or greater). Photographs of the DSW were taken at each dressing change following removal of the dressing. Surgeons were therefore partially blinded to dressing type when assessing re-epithelialisation, within the limits described above. Krippendorff’s-α on these data was 0.805, indicating a reliable correlation between the three surgeons’ assessments of days to re-epithelialisation [22, 23]. There were no significant differences in time to re-epithelialisation when direct observation and clinical decision-making, as determined by no ongoing need for dressings, were compared to photographs and independent scoring. The median time to re-epithelialisation was 7 days, with an IQR of 7–10 days. An inverse transformation was performed to normalise the ‘days to re-epithelialisation’ data for subsequent analyses.

With co-variates included, there was no statistically significant difference across the three dressings with respect to time to re-epithelialisation (ANCOVA; *p* = 0.0678).

Multiple linear regression (by age at surgery, sex, and median thickness of a central biopsy of the STSG) demonstrated younger patients had DSWs that re-epithelialised more rapidly (*p* = 0.0207).

#### Pain

The majority of patients had pain scores of zero at each time point and with each scale (NRS, FLACC, FPS-R). Because of this, pain was dichotomised to ‘no pain (score of zero)’ and ‘some pain’ for statistical analyses. NRS scores were done at 4 (61/90, 67.8% no pain) and 24 h (71/89, 78.9% no pain). FLACC scores were done at 4 h by trained staff in the postoperative care unit or on the ward (69/91, 85.2% no pain). NRS and FLACC scores were also done following removal of the DSW dressing at first dressing change.

There were no statistically significant differences in pain scores across the three DSW dressings. NRS at 4 (Pearson’s *χ*^2^, *p* = 0.891) and 24 h (Pearson’s *χ*^2^, *p* = 0.897), FLACC scores at 4 h (Pearson’s *χ*^2^, *p* = 0.777), and both NRS (Pearson’s *χ*^2^, *p* = 0.549) and FLACC (Pearson’s *χ*^2^, *p* = 0.788) at first dressing change showed no significant differences according to DSW dressing.

Regression analyses were performed using age at surgery, sex of patient, and median thickness of a central biopsy of the skin graft [15]. Logistic regression demonstrated a significant effect for sex at first dressing change (*p* = 0.019 for NRS, *p* = 0.024 for FLACC). Boys had lower pain scores at first dressing change; assessed by both their parents and by clinic staff. This effect was not seen at subsequent dressing changes.

Children over 3 years of age were asked to score their pain directly using the FPS-R. There were only 24 patients in this age range, too few for us to be able to draw any conclusions regarding differences across the three dressings used in this trial.

### Secondary outcome measures

#### Itch

Itch was assessed at the same time points as pain assessments were taken. The same methods were used to assess the results of itch scores. In children, the Itch Man Scale has been validated for those over 6 years, with parents being taken as a proxy for children under that age [17]. Itch scores were available at 4 and 24 h for 91/101 individual patients. The remaining patients were still intubated at 24 h post-STSG (10 patients). Only 7/91 (7.7%) patients or parents reported any itch at 4 h, and only 10/91 (11%) reported any itch at 24 h. The remaining patients had scores of zero for both time points. We therefore dichotomised this outcome to no itch (score of 0) vs some itch (score of 1–4) at these time points.

There were no statistically significant differences in itch across the three dressings at each time point (Pearson’s *χ*^2^, *p* = 0.835 at 4 h, *p* = 0.311 at 24 h, *p* = 0.799 at first dressing change).

Itch is one component of the patient component of the POSAS scale (NRS 1-10, see below for a fuller explanation) [19, 21]. Parents were used as a proxy for younger children. There was no statistically significant difference in dichotomised itch scores using this component of the POSAS at the 3 and 6-month reviews of patients (Pearson’s *χ*^2^, *p* = 0.125 at 3 months, *p* = 0.845 at 6 months).

Logistic regression adjusting for age at STSG, sex of patient, and thickness of STSG central biopsy was performed. This demonstrated a significant effect for age at surgery when measured at first dressing change *(p* = 0.005), with younger patients assessed as having more itch. This effect was not observed at any of the other assessments (4 and 24 h, other dressing changes, 3- and 6-month assessments).

#### Scarring

Scarring was assessed using the POSAS scale and by ultrasound measurement of scar thickness.

The POSAS scale contains patient and observer scales. Each of these scales assesses six elements (NRS 1-10), for a total score between 6 and 60. There is also an overall score (1–10) as a global assessment of scarring. In this trial, parents were used as proxies for younger children, and trained staff from the PLCBC performed the observer scale measurements.

##### Scarring at 3 months using POSAS

At 3-month review, 57 patients were available for scarring assessment. For 33 of these patients (58%), the total POSAS was under 10 (scale 6–60). Total POSAS was dichotomised to ‘less than 10’ and ‘10 or more’. The POSAS overall assessment (scale 1–10) scores from both observer and patient were also assessed.

There was no statistically significant difference across dressing type, when assessed by a trained clinical observer (Pearson’s *χ*^2^, *p* = 0.490).

Overall patient scores on the POSAS scale (1–10) were not significantly associated with any of the trial dressings (Pearson’s *χ*^2^, *p* = 0.3172).

Regression analysis demonstrated a significant effect related to thickness of the STSG harvested from the donor site. Thinner STSGs harvested were significantly associated with a lower overall patient POSAS score at 3 months (*p* < 0.0001). This association was not seen with the total observer POSAS score at 3 months (*p* = 0.106).

##### Scarring at 6 months using POSAS

At the 6-month review, 66 patients were available for scarring assessment. The total POSAS observer score was under 10 for 51 children (77.3%). The total POSAS patient score was under 10 for 34 children (51.5%). Scores were again dichotomised as at 3 months.

There was no statistically significant difference across dressing type at 6 months when assessed by a trained clinical observer (Pearson’s *χ*^2^, *p* = 0.075) or by the patients or parents themselves (Pearson’s *χ*^2^, *p* = 0.355). Multiple logistic regression demonstrated an association with the patient or parent’s overall global rating of the scar using the POSAS patient overall scale, and the thickness of the STSG taken. Thinner STSGs harvested from the donor site were associated with a lower (i.e., ‘better’) overall scar rating by the patient or parent at 6 months, nudging significance (*p* = 0.050). There were no other significant associations on regression analysis.

##### Sonographic SCAR thickness at 3 and 6 months

Ultrasound measurements of 43 DSWs were available at 3 months and 54 DSWs at 6 months. There was no significant association between ultrasound thickness of scarring at 3 or 6 months, and any of the three trial dressings (*p* = 0.8725 at 3 months, *p* = 0.9607 at 6 months).

Regression analysis showed a significant association between thinner scars sonographically following thinner STSGs harvested from those donor sites (as measured by a central biopsy of the STSG) [15]. This effect was seen at 3 months (*p* = 0.033), but not at 6 months (*p* = 0.487).

#### Cost

The three donor site dressings used in the trial are available in a variety of sizes in our hospital; as follows:

- Algisite™ M—5 × 5 cm, 10 × 10 cm, 15 × 20 cm
- Cuticerin™—7.5 × 7.5 cm, 7.5 × 20 cm, 20 × 40 cm
- Sorbact®—15 × 7.5 cm

Unit prices for the above dressings in Australia range from $0.40 to $12.42, depending on the size and type of dressing used. Approximate prices per centimetre squared are $0.038–$0.083 (Algisite™ M), $0.002–$0.007 (Cuticerin™), and $0.053–$0.107 (Sorbact®). Nursing times did not differ between the three dressings since they took similar times to apply and remove. As a percentage of the overall cost of care of these patients, these differences in cost are not significant. A whole dressing must be opened to cover a wound—so pack cost rather than centimetre squared comparisons must be considered in choosing which dressing to use.

There were no complications associated with any of the dressings, and therefore, no cost increases incurred through treating complications.

#### Ease of dressing application

This secondary outcome measure was abandoned early in the trial as it proved too difficult to quantify, given the confounder of the range in sizes of the DSWs.

#### Adverse effects

There were no adverse effects related to the DSW dressing with any patient.

## Discussion

### Motivation

This pragmatic trial was designed to determine which of the three DSW dressings currently in use in our children’s burns centre was the best performing. We found no significant differences in any of our pre-specified outcomes.

This is the first RCT in DSW dressings to include depth of the STSG harvested as a co-variate. We found STSGs harvested using a powered calibrated dermatome are not as uniform as has previously been assumed [15]. Our published data demonstrate no significant differences in mean STSG biopsy thickness across surgeons, patient age or sex, freshness of dermatome blade (first pass, second pass, etc.), or how far into the operation each pass was taken (as a measure of possible surgeon fatigue) [15]. Importantly, each pass is independent of neighbouring passes—a prior ‘thick’ STSG does not predict the next will be similarly thick. Within this cohort, our data demonstrated only 50% of STSGs harvested had a median thickness within two thousandths of an inch of the dialled thickness of 0.007 in. (0.005–0.009 in.) [15]. We found significant differences between our three trial arms in DSW area and mean STSG thickness. There were no significant differences regarding sex or age. Once these co-variates were included in the analysis, any apparent differences in primary or secondary outcomes disappeared. It is possible previous dressing trials using DSWs as their test bed may suffer from the same confounding variables, meaning apparent differences in outcomes could be explained with reference to donor site area, and/or thickness of the STSG harvested.

### Limitations

This trial is limited to a paediatric population with small- to medium-sized DSWs. In cases with larger DSWs, patients were often still intubated at 24 h, meaning pain data were not available and thus these results may not apply to adults and/or larger DSWs.

We did not record Fitzpatrick phototypes for our patients, so it is possible this is a confounding variable. However, recent data from Western Australia demonstrate no differences in scar outcomes following burns in Fitzpatrick 1–3 children when compared to Fitzpatrick 4–6 children [24].

Our data capture rates for primary outcomes were good, with enough patients in each arm to reach power for primary outcomes. Our pain data capture for dressing change was not as complete as it was for early measurements. Re-epithelialisation assessment was potentially confounded by being only partially blinded to trial arm.

Due to our dispersed patient population, not all patients were able to attend for their 3- or 6-month assessments, and so there are missing data in these groups. There are some data to suggest a thinner STSG leads to a better looking DSW at 3 months, across multiple modalities. This was not sustained at 6 months.

We chose to measure a single biopsy from the centre of the STSG, in order to determine the thickness of the actual STSG (as opposed to the dialled thickness on the dermatome). It is possible this biopsy is not reflective of the totality of the STSG taken, or that thickness was altered during processing. However, this is the first study that does not assume all STSGs are uniform and instead to include a measured thickness as a co-variate in analyses.

### Generalisability

Over the course of this trial, 131 patients were booked for STSG. In five cases, the procedure was cancelled at preoperative review. Our population is widely dispersed geographically, and our practice is to book these patients for review and STSG based on photographs or prior dressing change. This potentially saves a visit, but risks procedures being cancelled on the day. There were 10 ineligible patients. Of the 116 remaining patients, 6 were missed and 9 declined to participate. Thus, 101 of 116 potentially eligible patients (87%) took part in the trial. This trial therefore represents the majority of patients requiring grafting in our centre, limiting the potential for a biased selection of patients and increasing the generalisability of these results to our patient population.

Pre-infiltration with local anaesthetic prior to STSG harvest is a common technique among surgeons, but we chose not to do so in our trial to further standardise our DSWs. We were concerned pre-infiltration might confound our STSG thickness measurements and so chose topical local anaesthetic instead. Topical local anaesthetic has been shown to give good DSW analgesia in small series [25–27]. Our results support these previous publications.

Alginates are the most common DSW dressing used worldwide [3, 4, 28]. This trial uses alginates as an active control, against which two other dressings were compared. Within the limits of this trial, there are no significant differences between these three dressings.

### Interpretation

This trial demonstrates no superior dressing for short-term pain, itch, and re-epithelialisation outcomes among the three dressings tested. It also demonstrates no superior dressing among the three used with respect to longer-term scarring up to 6 months. These results are consistent with our pre-trial subjective opinions. Time to re-epithelialisation, pain and itch scores, and scarring within the trial are consistent with assessments from out-of-trial patients in our database both before and after this RCT. There were no adverse effects observed with any of the dressings used, in particular, infection and its possible effect on delaying healing.

Given these findings, and the fact that the unit cost of these dressings is minor when compared to the other costs of care for these patients, there is no economic argument to switch from one dressing to another. This is particularly so given the range of sizes available. As a result of the trial, surgeons in the unit utilise different DSW dressings depending on size of the DSW or its site.

Regression analyses of our data demonstrate shorter DSW re-epithelialisation times in younger patients. This result is consistent with previous studies, dating back as far as the First World War [29]. Longer re-epithelialisation times in older patients may potentially unmask differences between the three dressings in use; differences that are not seen in more rapidly healing children.

Regression analysis also demonstrated decreased pain in males at the first dressing change and decreased itch in younger patients at the first dressing change. This was not the case with subsequent dressing changes. These data should be interpreted with caution, as they are not consistently demonstrated across multiple dressing changes. The scales used often had parents standing in as proxies for their children. Validation data for younger children, and the use of parents as proxies, are lacking for these scales. It is possible that what is being measured here is an expectation effect, rather than a true difference.

This is the first RCT in donor site dressings that has included the thickness of the harvested STSG as a co-variate. Contrary to opinion, DSWs are not uniform and their depth varies greatly. Data from our unit demonstrate only 50% of DSWs harvested at 0.007 in. will fall between 0.005 and 0.009 in. when a central biopsy of the graft is measured [15]. This RCT showed better early scarring on those DSWs where a thinner STSG was harvested. This effect was seen across multiple modalities, POSAS, and ultrasound of scar thickness.

## Conclusions

In a paediatric population, there are no significant differences noted when comparing Algisite™ M, Cuticerin™, and Sorbact® as DSW dressings following STSG. Time to re-epithelialisation, and pain scores, are not significantly different. Itch during and after re-epithelialisation, and scarring at 3 and 6 months, are not significantly different when measured by staff or carers of these children using the POSAS and Itch Man Scales. Ultrasound assessment of scar thickness is not significantly different at 3 and 6 months. This trial demonstrates no imperative to alter our current practices.

## Abbreviations

ANCOVA: Analysis of co-variance
ANOVA: Analysis of variance
CCBTR: Centre for Children’s Burns and Trauma Research
CONSORT: Consolidated Standards of Reporting Trials
DACC: Dialkylcarbamoylchloride
DSW: Donor site wound
FLACC: Face, legs, activity, cry, consolability pain scale
FPS-R: Revised Faces Pain Scale
HREC: Human research ethics committee
MCID: Minimum clinically important difference
NRS: Numeric rating scale
PLCBC: Pegg Leditschke Children’s Burns Centre
POSAS: Patient and Observer Scare Assessment Scale
RCT: Randomised controlled trial
SD: Standard deviation
STSG: Split-thickness skin graft
